# Trastuzumab in patients with breast cancer and pre-existing left ventricular systolic dysfunction

**DOI:** 10.1186/s40959-024-00220-6

**Published:** 2024-03-15

**Authors:** Azin Alizadehasl, Mina Mohseni, Kamran Roudini, Parisa Firoozbakhsh

**Affiliations:** 1 Cardio-Oncology Research Center, Rajaie Cardiovascular Medical and Research Center, Iran University of Medical Sciences, Tehran, Iran; 2 Department of Internal Medicine, Hematology and Medical Oncology Ward, Cancer Research Center, Cancer Institute, Imam Khomeini Hospital Complex, Tehran University of medical Sciences, Tehran, Iran

**Keywords:** Trastuzumab, Left ventricular dysfunction, Cardiotoxicity, Targeted therapy

## Abstract

**Background:**

Trastuzumab is one of the most effective treatments in HER-2 positive breast cancer patients. However, it is associated with development of cardiomyopathy/heart failure (HF) which is often a limiting side effect and associated with overall adverse outcomes. As a result, patients with pre-existing LV systolic dysfunction (LVSD) are often excluded from receiving anti-HER-2 therapy, which may lead to less effective cancer treatment and worse prognosis.

**Objectives:**

The current study aims to evaluate the safety of trastuzumab in patients with HER-2 positive breast cancer and pre-existing LVSD.

**Methods:**

In this retrospective cohort study, 36 consecutive patients at a single center in Iran with HER-2 positive breast cancer with asymptomatic mild LVSD with LVEF 40–53% without heart failure symptoms and those who were closely followed in the Cardio-Oncology clinic before initiating the treatment and then every two cycles of trastuzumab were included. As per the program standard protocol they received a beta-blocker (carvedilol) and ACE-I (Lisinopril), up to the maximum tolerated dose, if there were no contraindications. Patients underwent routine echocardiography with global longitudinal strain (GLS) assessment every 3 months per guideline recommendations and were followed up 6 months after the end of treatment. Primary composite outcomes included myocardial infarction (MI), cardiac arrhythmia, heart failure(HF) symptoms and cardiovascular death. Secondary outcome was ≥ 10% reduction in LVEF or ≥ 15% reduction in GLS compared to baseline. If the LVEF decreased below 40%, the treatment was temporarily interrupted for one or two cycles, and spironolactone was added to the patient’s treatment. If the LVEF improved ≥ 40%, trastuzumab was rechallenged. Data analysis was performed using IBM SPSS Statistics 24.0. Software. Patients’ characteristics were reported using descriptive statistics, and its association with drop in LVEF or GLS was assessed using Pearson chi-square or Mann-Whitney U test. A *p*-value of less than 0.05 was considered significant.

**Results:**

Thirty-six patients were included in the study. Primary composite outcome was noted in 1(2.8%) patient. LVEF reduction of ≥ 10% occurred in 6(16.7%) of the patients, and a GLS reduction of more than 15% was detected in 4 (11.1%) of the patients. There was a significant association between a ≥ 10% reduction in LVEF and baseline systolic blood pressure (*P*-value: 0.04). LVEF reduction below 40% was observed in 3 (8.3%) patients, where trastuzumab was interrupted. All of these three patients had obesity (Median BMI 34.11, IQR 9.12) and uncontrolled HTN, and one of them had symptoms of heart failure (NYHA class II), for whom the trastuzumab treatment was discontinued. Among two patients, after the temporary interruption of trastuzumab, and addition of spironolactone, LVEF improved to above 40%, and the treatment was restarted with close cardiac monitoring; therefore, they could complete the entire one-year treatment period.

**Conclusions:**

Treatment with trastuzumab seems to be safe in patients with pre-existing LVSD (LVEF = 40–53%). Such high-risk patients should be strictly monitored and cardiovascular risk factors, such as HTN should be regulated.

## Introduction

Cancer remains a significant global health challenge and a leading cause of mortality, with GLOBOCAN 2020 reporting 19.3 million new cases and nearly 10 million deaths in 2020 alone. Among these, breast cancer emerges as the most frequently diagnosed cancer among women, ranking as the fifth leading cause of cancer-related deaths globally [1–3]. 

Concurrently, cardiovascular diseases have surfaced as a major cause of mortality and morbidity among cancer patients, particularly affecting female breast cancer survivors, for whom it is the primary cause of death. The advancements in breast cancer treatments have notably enhanced survival rates, albeit at the cost of increased exposure to the long-term side effects associated with anti-cancer drugs. Specifically, traditional chemotherapy and targeted therapies, such as anthracyclines and trastuzumab, have been linked to a heightened risk of cardiotoxicity, leading to various cardiac complications [4]. 

Breast cancers that overexpress the human epidermal growth factor receptor type 2 (HER-2) are known for their aggressive nature, higher metastasis rates, and poorer outcomes. However, the inhibition of HER-2 signaling in HER2-positive breast cancers has been a breakthrough, with trastuzumab (Herceptin) playing a pivotal role in improving patient outcomes. Clinical trials have validated the efficacy of trastuzumab in significantly lowering mortality and recurrence rates while enhancing survival, leading to its recommendation for both early-stage and metastatic HER-2 positive breast cancers [4–7]. 

Despite trastuzumab’s therapeutic success, its use is marred by cardiotoxicity risks, including heart failure and reduced left ventricular ejection fraction (LVEF), which can limit its application in breast cancer treatment. Although the incidence of severe heart failure post-trastuzumab therapy is relatively low, the risk of an asymptomatic decline in LVEF is significant. This has necessitated the development of strategies for early detection and management of trastuzumab-induced cardiotoxicity, incorporating cardiac biomarkers, imaging modalities, and preventive measures such as beta-blockers and angiotensin-converting enzyme inhibitors [5, 8, 9]. 

Historically, the occurrence of cardiotoxicity necessitated the suspension of trastuzumab therapy pending the resolution of LV dysfunction, a practice that risked cancer progression and poorer outcomes. Recent pilot studies, however, suggest that continuing trastuzumab therapy in asymptomatic patients with LVEF above 40% might be safe and could potentially improve patient prognosis and survival rates [10–14]. 

This study aims to assess the cardiac function of patients with asymptomatic LV dysfunction (LVEF > 40%) prior to initiating trastuzumab therapy in a retrospective cohort. With the consensus that no superior alternative treatments exist, these patients were continued on trastuzumab with vigilant cardiac monitoring, as advised by their oncologists.

## Materials and methods

### Study design & population

This single-center retrospective cohort study was conducted based on the recordings of the patients who were referred to the Cardio-Oncology clinic of Rajaie Cardiovascular Medical and Research Center from 2017 to 2022 and had their data registered in the Iranian Cardio-Oncology Registry [15]. Patients were included in the current study if they met the following inclusion criteria:

1. Female HER-2 positive breast cancer patients, regardless of their cancer stage, and their data were registered in the Iranian Cardio-Oncology Registry.
2. Candidates of receiving trastuzumab, as the best available cancer treatment.
3. LVEF 41–53% at the baseline, prior to initiation of trastuzumab therapy. The cause of cardiomyopathy in all patients was their previous chemotherapy.
4. Being followed up for 6 months after the end of the treatment.

Patients were excluded from the study, if:

1. They had symptoms of heart failure at the baseline, prior to initiation of the treatment, or had a previous history of hospitalization due to heart failure.
2. They were candidates for concurrent chemotherapy.
3. The symptoms of heart failure, or reduced LVEF < 40% occurred following the trastuzumab treatment, and didn’t improve despite interruption of trastuzumab and receiving spironolactone.

### Patient management &interventions (Add a flow diagram to describe how patients were selected and followed for easier visual)

Patients were visited in the cardio-oncology clinic, prior to initiating trastuzumab and before every two cycles of treatment.

The visit of patients included taking a full history which means patient’s demographic data, past medical history (diabetes, hyperlipidemia, hypertension), habitual history (cigarette smoking, consumption of alcohol, opium, or any other drugs), drug history, and previous history of receiving chemotherapy, HER-2-targeted therapy, hormone therapy, current or previous radiotherapy. Moreover, a complete physical examination, electrocardiogram (ECG) and advanced echocardiography were performed.

**Fig. 1:**
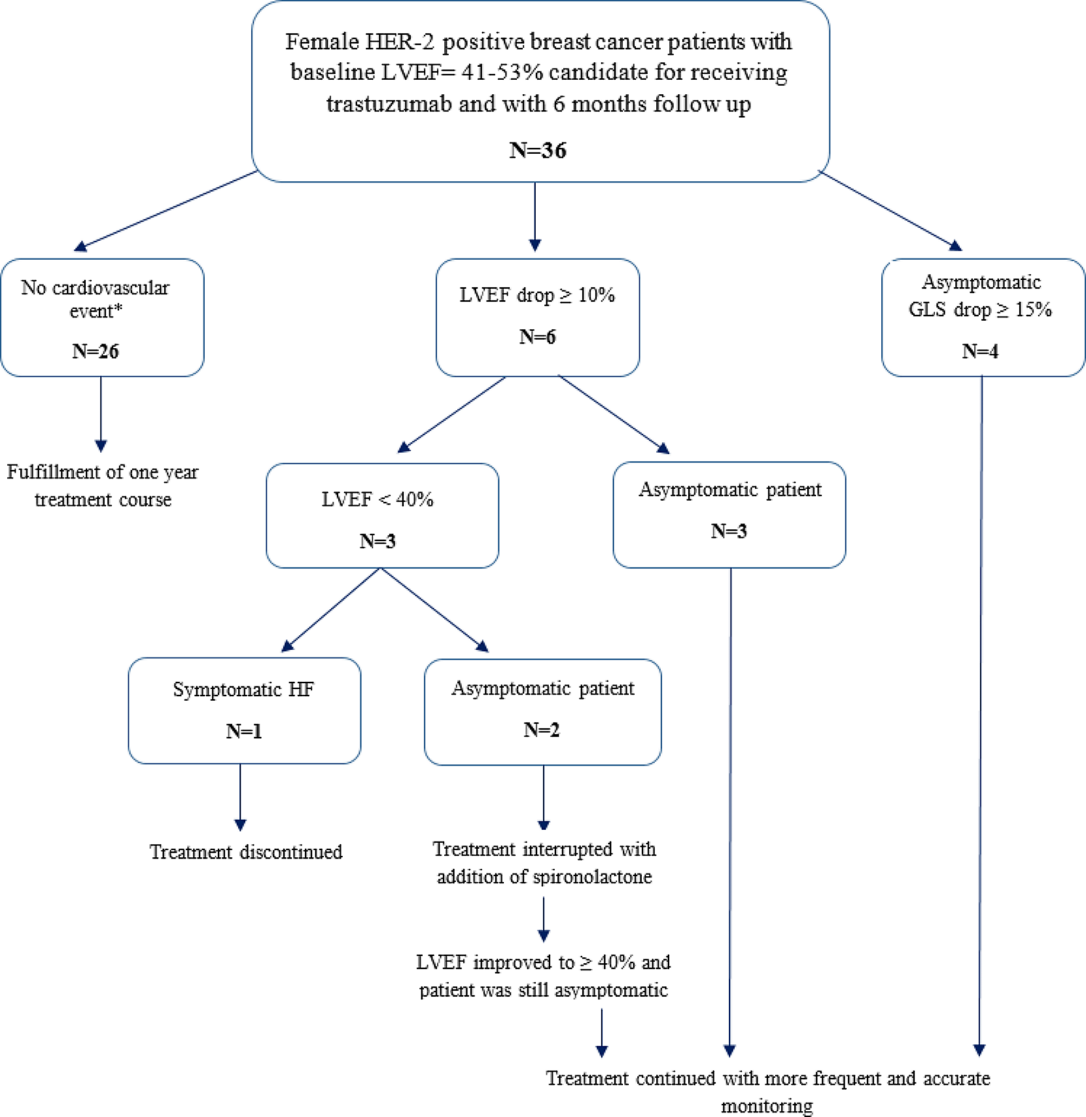
Patients inclusion and follow up flowchart. HER-2: human epidermal growth factor receptor type 2, LVEF: left ventricular ejection fraction, GLS: global longitudinal strain. *Cardiovascular event was defined as any cases of myocardial infarction, cardiac arrhythmia, heart failure symptoms and cardiovascular death

At first, AC chemotherapy regimen (60 mg/m2 adriamycin and 600 mg/m2 cyclophosphamide on day 1 and then every 21 days for 4 cycles was prescribed for these patients. Because the patients’ LVEF was normal before the start of anthracycline treatment, dexrazoxane was not used. Treatment was continued using 80 mg/m^2^ paclitaxel weekly for 12 weeks, and 4 mg/kg trastuzumab as the first dose, started concurrently with the first dose of paclitaxel, followed by 6 mg/kg trastuzumab every 21 days until the fulfillment of one entire year. The selected patients also received standard heart failure treatment with beta-blocker (carvedilol) and ACE-I (Lisinopril) in the absence of contraindications, up to the maximum tolerated dose. Patients were followed up 6 months after the end of treatment.

any case of myocardial infarction, cardiac arrhythmia, symptomatic heart failure, or death due to Cardiovascular disease occurred during the treatment, was recorded as a cardiac event and the treatment was stopped. In case of asymptomatic drop ≥ 10% in LVEF, or ≥ 15% in GLS (global longitudinal strain) [16], trastuzumab therapy was continued with more frequent and more accurate monitoring. By dropping LVEF to below 40%, the treatment was interrupted temporarily for one or two cycles, and spironolactone was added to the patient’s treatment. If LVEF improved (LVEF ≥ 40%) and the patient was still asymptomatic, the treatment was restarted. Otherwise, the patient was excluded from the study and registered as a cardiac event. In cases of symptomatic heart failure, trastuzumab was stopped and the patient was recorded as a cardiac event. (Fig. 1).

### Study outcome

The efficacy outcome was the number of patients who were able to complete the trastuzumab cycles for one year of treatment. Primary composite outcomes included myocardial infarction (MI), cardiac arrhythmia, heart failure(HF) symptoms and cardiovascular death. Secondary outcome was ≥ 10% reduction in LVEF or ≥ 15% reduction in GLS compared to baseline.

### Statistical analysis

Data analysis was performed using IBM SPSS Statistics 24.0. software (IBM Corp. Released 2016. IBM SPSS Statistics for Windows, Version 24.0. Armonk, NY: IBM Corp.). Patients’ characteristics were reported using descriptive statistics. Continuous variables were presented as mean ± SD, and categorical variables were presented as numbers (percentages). In order to assess the association of drop in LVEF or GLS, and patients’ characteristics, Pearson chi-square or Fischer’s exact test was used for categorical variables, and the Mann-Whitney U test was used for numerical variables. A *p*-value of less than 0.05 was considered significant.

## Results

Thirty-six patients met the inclusion criteria. The mean age of the patients was 53.19 ± 12 years, with a minimum age of 30 years and a maximum of 84 years. 72.2% of the patients were overweight or obese, 11.1% of them had diabetes, 19.4% had hypertension, 22.2% had hyperlipidemia, and 5.6% had coronary artery disease. 16.7% of the patients had metastatic breast cancer. 77.8% of patients had received radiation. Baseline background characteristics of the patients are demonstrated in Table 1.

**Table 1:** Baseline background characteristics of the participants

Variable	Descriptive statistics
Age (years), mean (SD)	53.19 (12.73)
BMI (kg/m²), mean (SD)	27.65 (4.39)
BMI Category, n (%)	
Normal	10 (27.8%)
Overweight	17 (47.2%)
Obese	9 (25%)
Heart rate (bpm), mean (SD)	86.3 (11.2)
Systolic BP, mean (SD)	121.4 (15.9)
Diastolic BP, mean (SD)	77.7 (9.3)
Past medical history, n (%)	
Diabetes mellitus	4 (11.1%)
Hypertension	7 (19.4%)
Hyperlipidemia	8 (22.2)
Coronary artery disease	2 (5.6)
Cancer details	
Laterality of breast cancer, n (%)	
Right breast	14 (38.9%)
Left breast	22 (61.1%)
Receptor status, n (%)	
ER-Positive	19 (52.8%)
PR-Positive	23 (63.9%)
Presence of metastasis, n (%)	6 (16.7%)
Previous radiation therapy, n (%)	28 (77.8%)
Positive family history of breast cancer, n (%)	15 (41.7%)

BMI: body mass index, SD: standard deviation, bpm: beats per minute, BP: blood pressure, ER: estrogen receptor, PR: progesterone receptor

**Fig. 2:**
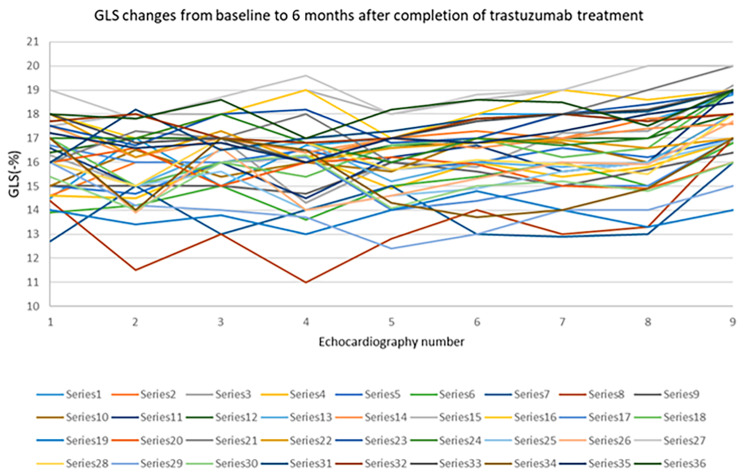
GLS changes from baseline to 6 months after completion of trastuzumab treatment. GLS: global longitudinal strain

**Fig. 3:**
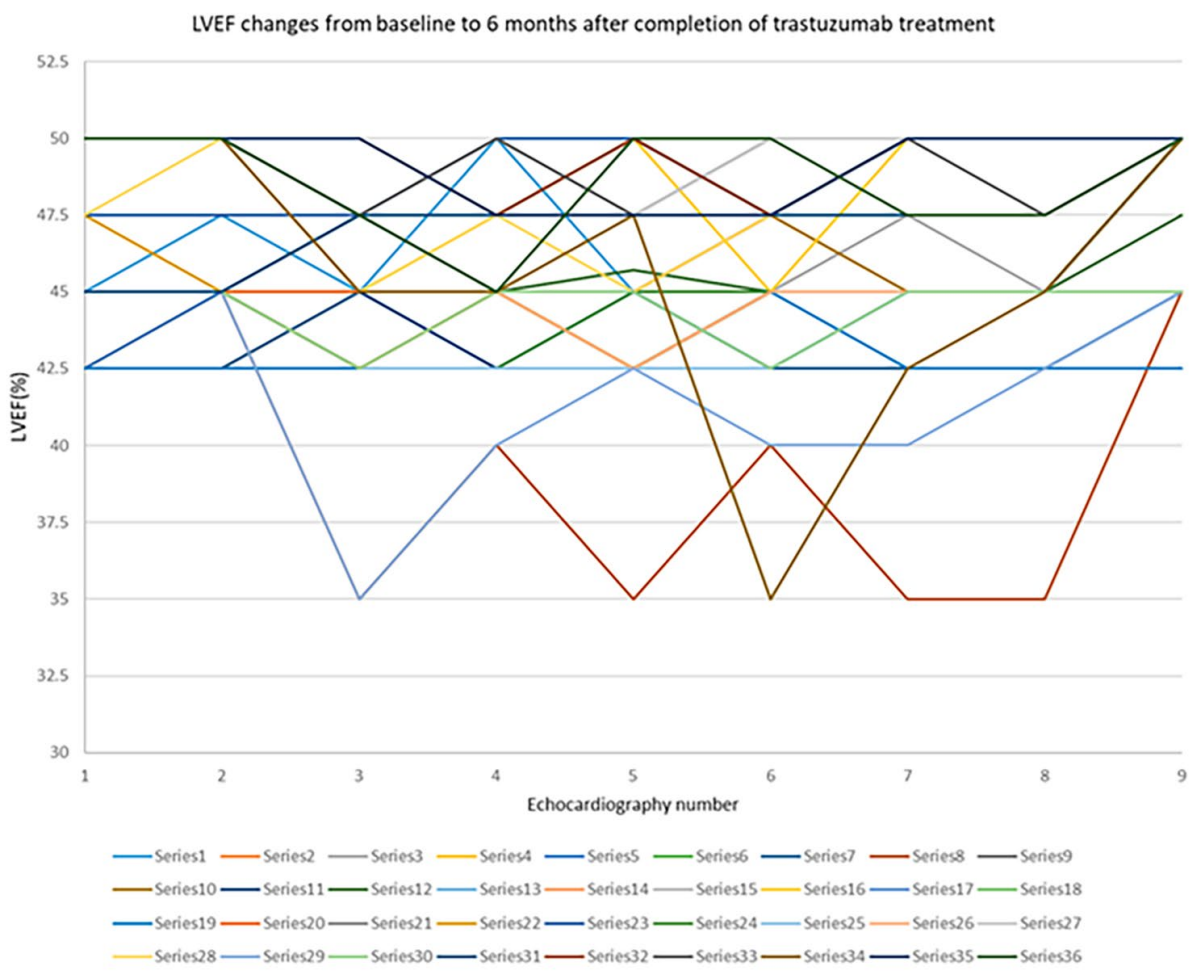
LVEF changes from baseline to 6 months after completion of trastuzumab treatment. LVEF: left ventricular ejection fraction

LVEF reduction of more than 10% occurred in 16.7% of the patients, and GLS reduction of more than 15% occurred in 11.1% of them (Figs. 2 and 3). However, when LVEF remained above 40%, the treatment was continued, and if LVEF dropped to below 40%, the treatment was interrupted.

There was no significant association between ≥ 10% LVEF reduction, and ≥ 15% GLS reduction and previous history of diabetes, hypertension, hyperlipidemia, and coronary artery disease. But more than 10% drop in LVEF had a significant association with baseline systolic blood pressure (*P*-value: 0.04) (Table 2).

**Table 2:** Predictors of decline in LVEF and GLS following treatment with Trastuzumab

	≥ 10% decline in LVEF				≥ 15% decline in GLS		
	Yes	No	*P*-Value		Yes	No	*P*-Value
BMI Category							
Normal	1 (10%)	9 (90%)	0.76		2 (20%)	8 (80%)	0.53
Overweight	3 (17.6%)	14 (82.4%)			1 (5.9%)	16 (94.1%)	
Obese	2(22.2%)	7 (77.8%)			1 (11.1%)	8 (88.9%)	
Baseline heart rate, median (IQR)	88.5 (26.75)	90 (12.5)	0.564		82.5 (27.75)	90 (12.5)	0.33
Baseline systolic blood pressure, median (IQR)	135 (44.5)	120 (15.75)	0.04		115 (17.5)	120 (19.75)	0.19
Baseline diastolic blood pressure, median (IQR)	85 (20.25)	80 (12.75)	0.22		82.5 (16.25)	80 (14.25)	0.38
Past medical history							
Diabetes mellitus	1 (25%)	3 (75%)	0.53		0	4 (100%)	0.61
Hypertension	3 (42.9%)	4 (57.1%)	0.07		1 (14.3%)	6 (85.7%)	0.59
Hyperlipidemia	2 (25%)	6 (75%)	0.4		1 (12.5%)	7 (87.5%)	0.65
Coronary artery disease	1 (50%)	1 (50%)	0.31		0	2 (100%)	0.78
Cancer details							
Laterality of breast cancer							
Right breast	3 (21.4%)	11 (78.6)	0.65		1 (7.1%)	13 (92.9%)	0.49
Left breast	3 (13.6%)	19 (86.4%)			3 (13.6%)	19 (86.4%)	
Receptor status							
ER-Positive	3 (15.8%)	16 (84.2%)	0.61		1 (5.3%)	18 (94.7%)	0.32
PR-Positive	4 (17.4%)	19 (82.6%)	0.63		1 (4.3%)	22 (95.7%)	0.12
Presence of metastasis	1 (16.7%)	5 (83.3%)	0.74		0	6 (100%)	0.46
Previous radiation therapy	3 (10.7%)	25 (89.3%)	0.1		3 (10.7%)	25 (89.3%)	0.65

LVEF: left ventricular ejection fraction, GLS: global longitudinal strain, BMI: body mass index, IQR: interquartile range, ER: estrogen receptor, PR: progesterone receptor

**Fig. 4:**
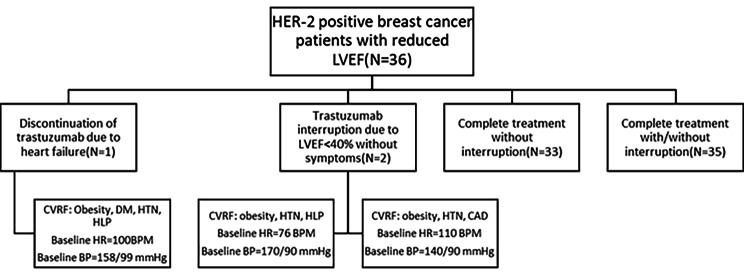
Flow diagram of final results. HER-2: human epidermal growth factor receptor type 2, LVEF: left ventricular ejection fraction, CVRF: cardiovascular risk factor, DM: diabetes mellitus, HTN: hypertension, HLP: hyperlipidemia, HR: heart rate, BPM: beats per minute, BP: blood pressure, CAD: coronary artery disease

One of the patients showed symptoms of heart failure (NYHA class II) and received the proper treatment for it. Although the symptoms were revealed, but the patient wasn’t rechallenged with trastuzumab due to the LVEF drop below 35%. This patient’s LVEF improved to 45% in follow-up echocardiography which was performed 6 months afterward. Two patients experienced a drop in LVEF to less than 40%, and trastuzumab treatment was temporarily interrupted and spironolactone was added to their treatment regimen. After interrupting one cycle of trastuzumab, LVEF increased to over 40%, and the treatment was continued. Other patients who had a ≥ 10% reduction in LVEF and ≥ 15% reduction in GLS were asymptomatic or symptomatic at the level of NYHA class I, despite the drop in LVEF and GLS. The characteristics of one patient who had to discontinue trastuzumab and two patients who had to interrupt trastuzumab are shown in Fig. 4. All three patients had a history of uncontrolled HTN, which seems to be an important factor in causing cardiotoxicity, and BMI of these three patients were above 30.

**Fig. 5:**
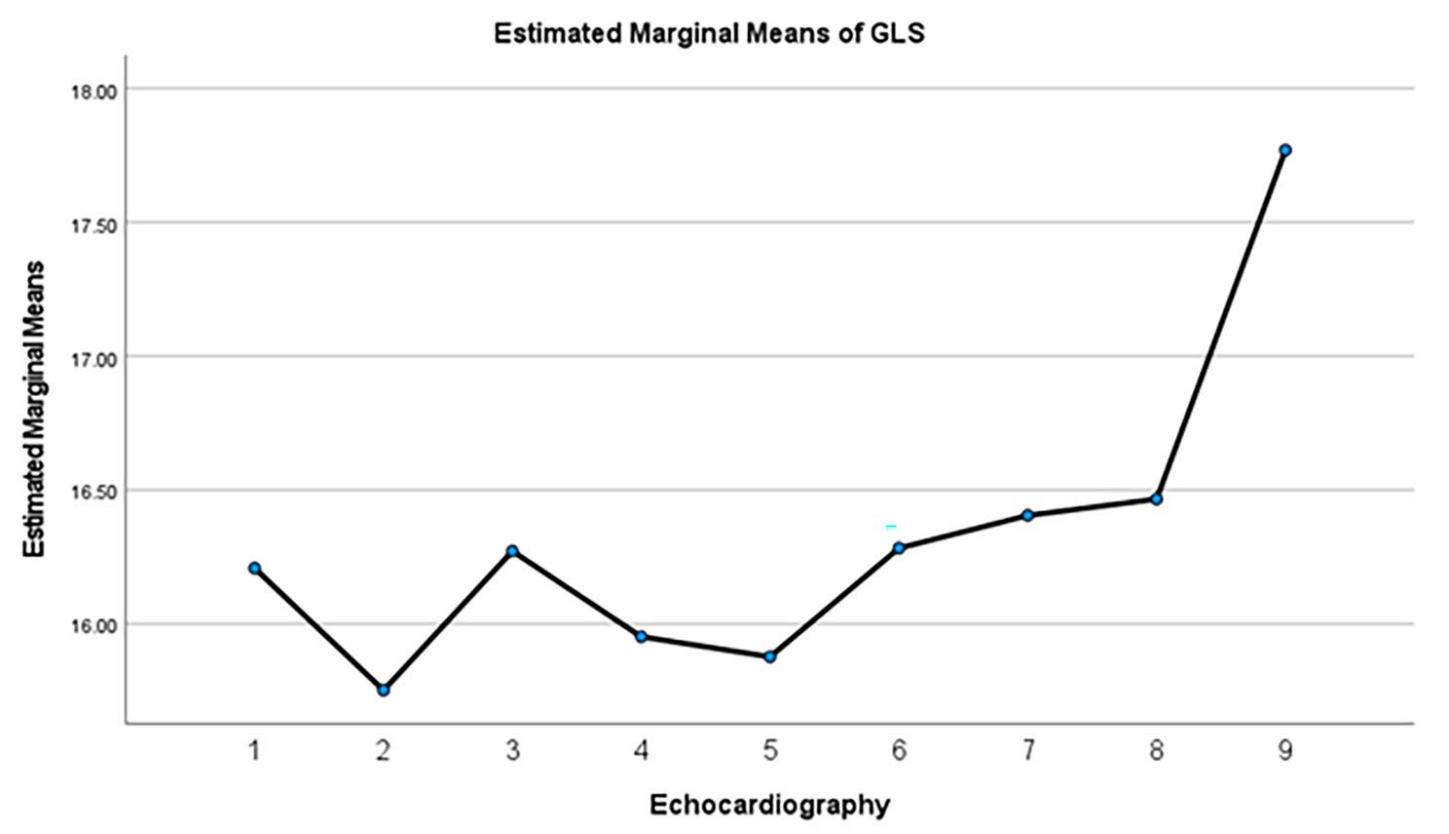
Estimated marginal means of GLS in HER-2 positive breast cancer patients following treatment with trastuzumab in serial echocardiography. Echocardiography 1 to 8 were performed during 12 months of trastuzumab treatment, and echocardiography 9 was performed 6 months after completion of treatment. GLS: global longitudinal strain

**Fig. 6:**
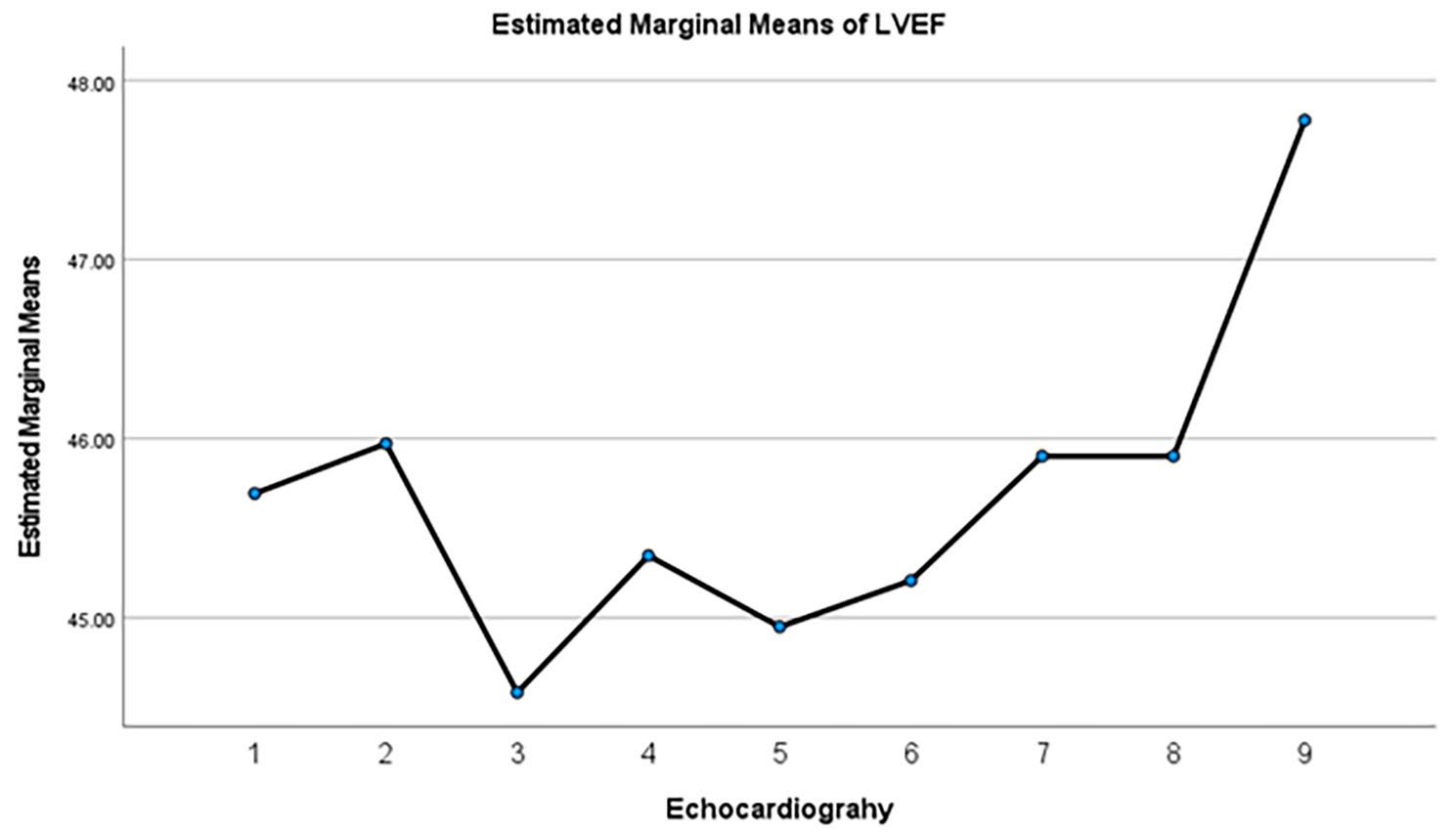
Estimated marginal means of LVEF in HER-2 positive breast cancer patients following treatment with Trastuzumab in serial echocardiography. Echocardiography 1 to 8 were performed during 12 months of trastuzumab treatment, and echocardiography 9 was performed 6 months after completion of treatment. LVEF: left ventricular ejection fraction

Most of the drops in mean GLS of the patients occurred in the second follow-up echocardiography (Fig. 5). The second follow-up echocardiography was performed between the third and fourth courses of trastuzumab treatment (Fig. 6). Most of the decrease in mean LVEF occurred in the third follow-up echocardiography, which was performed between the fifth and sixth courses of treatment.

## Discussion

HER-2 gene amplification and protein overexpression occurs in 15–20% of breast cancers. It is associated with a worse prognosis and a higher risk of cancer recurrence. The most important side effect of trastuzumab is cardiotoxicity, which often occurs as LV dysfunction [17]. In a study, 16.6% of patients who were treated with trastuzumab experienced cardiotoxicity. LVEF drop of more than 10% occurred in 15.1% and LVEF drop below 50% occurred in 5.9% of patients. Symptomatic heart failure with NYHA class II occurred in 4.5% and NYHA class 3, 4 in 0.5% of patients. Discontinuation of trastuzumab therapy due to cardiac causes occurred in 3.8% of patients. No treatment-related death was reported [6]. In our study population, LVEF reduction of more than 10% occurred in 16.7% of patients and GLS reduction of more than 15% occurred in 11.1% of patients. There were no death records, and symptomatic heart failure of NYHA class II occurred in only one patient.

According to the recommendations of the authors of the European Society for Medical Oncology (ESMO), in case of mild asymptomatic cardiac dysfunction, along with the initiation of cardioprotective Therapies, it is better to continue treatment with trastuzumab [18]. 

A prospective phase I trial examined patients who were treated with trastuzumab but had an LVEF between 40% to the lower limit of normal or a drop in LVEF greater than 15% from baseline and were asymptomatic. The patients were treated with beta-blocker and ACE-I, and were followed-up and underwent serial echocardiography in the cardio-oncology clinic. In the study of these 20 patients, 18 patients completed the trastuzumab treatment without cardiac events. Two patients had a drop in LVEF below 40%, which improved after stopping the treatment. According to the results of this study, it may be possible to continue trastuzumab in the presence of mild cardiotoxicity as long as they are under close observe of a Cardio-Oncologist with the initiation of cardioprotective drugs including beta-blocker and ACE-I [11]. In our study, 3 patients had LVEF drop below 40%. With the temporary interruption of trastuzumab and the continuation of treatment with BB and ACE-I and the start of spironolactone for them, LVEF improved in two of them and they completed the entire course of trastuzumab therapy. One out of these three patients had NYHA class II symptoms and her treatment was discontinued. All three patients who experienced a drop in LVEF below 40% were obese, which require more attention and investigations in future studies.

In the SAFE-HEaRt study, patients with stage 1 to 4 HER2 positive breast cancer were candidates for treatment with trastuzumab, pertuzumab or ado-trastuzumab and had LVEF between 40 and 49% (without symptoms of heart failure). All patients were treated with beta-blocker and ACE-I if there were no contraindications. 30 patients were included in the study, and among them, 27 completed the full course of treatment. Two patients had cardiac events and one patient had a drop in LVEF below 35%. This study showed that the continuation of treatment in these patients along with careful cardiac monitoring and cardioprotective drugs can be safe [12]. It is noteworthy to mention that in our study, patients did not experience heart failure symptoms, despite the decreased LVEF, before the start of anti-HER-2 treatment.

In the long-term follow-up of 3.5 years, the continuation of HER2-targeted therapies in breast cancer patients with reduced LVEF along with cardioprotective drugs and careful cardiac monitoring sessions seems to be safe. Delayed occurrence of cardiac dysfunction is uncommon, and only one cardiac event occurred and, no death was reported. These results highlight the importance of collaboration between cardiologists and oncologists to provide the best treatment for this special population [10]. The continuation of cardioprotective treatments, including BB and ACE-I, can be beneficial for these patients both during treatment and after completing the course of treatment with trastuzumab, and it can even gradually improve the cardiac function of patients. In our study, the patients were followed up for 6 months after the completion of trastuzumab therapy. Mean GLS and LVEF were improved on the follow-up echocardiography which were performed 6 months after completion of course of treatment. (Figures 5 and 6) Our finding shows the reversibility of TIC in most patients.

In a retrospective review study that examined the safety of continuing trastuzumab in patients with left ventricular dysfunction, 18 patients were examined. 17 out of 18 patients with mildly reduced LVEF (as a complication of trastuzumab treatment) and mild symptoms of heart failure continued anticancer treatment with no death records or serious cardiac complications. cardioprotective treatments including BB and ACE-I were provided for all patients [14]. In our study, there was no death and 35 out of 36 patients completed the course of trastuzumab treatment.

Nowsheen et al.‘s study showed that breast cancer patients who have LV dysfunction at baseline can also receive trastuzumab. They showed that compared to the control group (normal LVEF), the incidence of LV dysfunction was not different, however the risk of heart failure is higher in such patients. These patients must be carefully monitored by a cardiologist or cardio-oncologist. In Nowsheen et al.‘s study, LVEF drop of more than 10% had no significant relationship with cardiovascular risk factors such as hypertension, DM and hyperlipidemia, which was consistent with our study. However, in our study uncontrolled baseline blood pressure had a significant relationship with the drop in LVEF, which shows the importance of controlling cardiovascular risk factors including blood pressure. It should be mentioned, due to the small sample size, these findings need to be interpreted with caution [19]. 

Global longitudinal strain (GLS) changes are more sensitive than LVEF parameter for diagnosis of cardiotoxicity due to cardiotoxic anticancer treatments. GLS is an excellent parameter to detect subclinical changes in cardiac function [20]. In our study the decrease in GLS preceded the decrease in LVEF, which shows that the decrease in GLS can be a good predictor for the decrease in LVEF.

### Study limitations

The main limitation of the current study that makes our findings less generalizable is our small sample size. It is also unfortunate that in this study, patients were followed up with echocardiography only for 6 months after the end of the treatment. We recommend that in the future, studies with a larger sample size and a longer follow-up period should be conducted in patients with LVEF above 40%. the other limitation of this study is the lack of a control group. As a result, we cannot compare the results of our study with a group of patients with normal LVEF.

## Conclusions

Use of trastuzumab as a HER2 inhibitor targeted therapy among select patients with mild asymptomatic cardiomyopathy (LVEF 40–53%) seems to be safe with close monitoring of cardiotoxicity and appropriate cardioprotective treatments (BB, ACE-I, /ARB, spironolactone). A past medical history of HTN, especially uncontrolled HTN, prior to starting trastuzumab can increase the risk of cardiotoxicity.

Perspectives: Continuing the treatment with trastuzumab seems to be safe in patients with reduced LVEF (40% or more). Strict control of cardiovascular risk factors, especially HTN, is recommended. It is recommended in future studies to investigate the safety of trastuzumab treatment in patients with LVEF below 40%.

## Data Availability

Data can be provided upon request.

## Abbreviations

HER-2: Human epidermal growth factor receptor type 2
LVEF: Left ventricular ejection fraction
LV: Left ventricle
ACE-I: Angiotensin-converting enzyme inhibitors
MI: Myocardial infarction
HF: Heart failure
GLS: Global longitudinal strain
HTN: Hypertension
NYHA Functional Classification: New York Heart Association Functional Classification
TIC: Trastuzumab-induced cardiotoxicity
BB: Beta blockers
ECG: Electrocardiogram
AC chemotherapy regimen: Adriamycin and cyclophosphamide chemotherapy regimen
SD: Standard deviation
ESMO: European Society for Medical Oncology
DM: Diabetes mellitus
ARB: Angiotensin receptor blockers
